# Neurological Involvement in Primary Systemic Vasculitis

**DOI:** 10.3389/fneur.2019.00430

**Published:** 2019-04-26

**Authors:** Shanshan Zhang, Dongli Yuan, Ge Tan

**Affiliations:** ^1^Department of Neurology, The First Affiliated Hospital of Chongqing Medical University, Chongqing, China; ^2^The Institute of Medical Information, Chongqing Medical University, Chongqing, China

**Keywords:** primary systemic vasculitis, central nervous system, peripheral nervous system, neurological involvement, clinical manifestation

## Abstract

Primary systemic vasculitis can affect every structure in both the central and peripheral nervous system, causing varied neurological manifestations of neurological dysfunction. Early recognition of the underlying causes of the neurological symptoms can facilitate timely treatment and improve the prognosis. This review highlights the clinical manifestations of primary systemic vasculitis in the nervous system.

## Introduction

Primary systemic vasculitis (PSV) can be defined as a heterogeneous group of uncommon diseases characterized by blood vessel inflammation and necrosis. Unlike secondary systemic vasculitis, which usually results from infections, connective tissue diseases, neoplasms, and drugs, PSV is considered to occur with no identified etiology (1). Nevertheless, recent researches have demonstrated that PSV might be associated with farming (2), silica exposure (2, 3), solvent exposure and hydrocarbons (2, 4, 5), allergy and family history of atopy (2, 6). Despite of unknown causes, two mechanisms, deposition of immune complexes and cell-mediated immunity, were found to possibly participate in the pathophysiology of PSV by causing immunological inflammation and necrosis of the vessel wall (7). And according to the classification criteria revised in 1990 by The American College of Rheumatology (8) and in 1994 and 2012 by the Chapel Hill Consensus Conferences (9, 10), the classification of PSV is based primarily on the major size of the affected vessels, although these disease-affected arteries may have overlaps in diameter.

Many neurologists have limited knowledge of PSV because most of these patients are treated by rheumatologists. However, both the central nervous system (CNS) and peripheral nervous system (PNS) are major targets in PSV and may become involved in the earliest stages; thus, patients may be referred to a neurologist first. Since the clinical manifestations of PSV are often non-specific, the differential diagnosis may be challenging.

CNS damage was reported to occur in 24% of cases with PSV, commonly due to antineutrophil cytoplasmic antibody (ANCA)-associated vasculitis and polyarteritis nodosa. The range of clinical expressions of PSV's CNS damage is relatively wide, including cerebrovascular manifestations such as hemorrhagic and ischemic stroke caused by intra/extra-cerebral vascular stenosis, aneurysm, and sinus venous thrombosis, meningeal and brain parenchymal involvement resulted from granulomatosis and perivasculitis, and encephalopathy due to cytokine damage.

Compared with the frequency of central vasculitic involvement, vasculitic peripheral neuropathies are more common and have been reported to occur in 37% of patients with PSV (11), and 25% of patients experience symptoms of peripheral neuropathy at initial presentation (12). Generally, vasculitic peripheral neuropathies result from the inflammation of precapillary arteries in the nerves, such as eosinophilic granulomatosis with polyangiitis (60–80% of patients), polyarteritis nodosa (50–100% of patients), granulomatosis with polyangiitis (Wegener's granulomatosis, 13–26.6% of cases), cryoglobulinemia (70% of cases), and microscopic polyangiitis (6–18% of cases), usually presenting in a uniform pattern such as multiplex mononeuropathy (accounting for 59–85% of cases) and symmetric polyneuropathy (41% of cases) (11–13). Nerve conduction studies have revealed a much higher frequency of peripheral neurological involvement than that in clinical case series and studies. Asymptomatic vasculitic neuropathy was detected in 21 of 270 cases (7.8%) of biopsy-proven vasculitis (14).

In addition, the introduction of nerve sonography, single-photon emission computed tomography (SPECT), magnetic resonance imaging (MRI), magnetic resonance angiography (MRA), magnetic resonance spectroscopy (MRS), and apparent diffusion coefficient (ADC) have greatly improved the diagnostic rate and accuracy of vasculitic neuropathy.

## Search Strategy

We performed an advanced literature search in PubMed and EMBASE for the period January 1, 1990, through September 1, 2017, using as a search query “systemic vasculitis” “nervous system.” Eligible studies were included according to the following criteria. Only human studies that were written in English were considered. All patients included in the studies were diagnosed according to the classification criteria defined by the American College of Rheumatology or the Chapel Hill Consensus Conference. Retrospective and prospective studies were included to determine the frequency of neurological manifestations. Case reports and case series were added in the reference list only when unusual features were described. Pediatric studies were excluded because the clinical features of children and newborns are different.

## Large-Vessel Vasculitis

Large vessels were defined as the aorta, vena cava, and their major branches. Large-vessel vasculitis is composed of Takayasu arteritis and giant cell arteritis.

### Takayasu Arteritis

Takayasu arteritis (TA) is a rare type of primary chronic inflammatory disease, causing stenosis, occlusion and aneurysm of arteries. TA mainly affects large-caliber arteries such as the aorta and its major branches in patients younger than 40 years old (more than 90% of patients are younger than 40), with a female predominance (female to male ratio is 10:1), and is more common in Asians (1). Studies have shown that more than half of patients with TA show neurological manifestations (15, 16) that range from dizziness/headaches to ischemic or hemorrhagic stroke.

Dizziness and headache are the most common complaints in patients with TA with neurological complications, accounting for ~78.1% (214/274) and 25.5% (70/274), respectively (16). However, headache and dizziness do not necessarily indicate CNS involvement. Visual disturbances affect 4.6–59.3% of patients, and 4–21.9% of TA patients with neurological manifestations were found to have syncope as the main manifestation.

Cerebrovascular complications have always been the focus of researchers. Transient ischemic attack accounts for 3–22.2% of the nervous system manifestations caused by TA. Stroke, as the most severe complication of TA, was found to occur in 10–20% of cases (16–18). Due to lack of knowledgement, the median and average delay between symptom onset and the diagnosis of TA are 2 years (19) and 52.4 months (16), respectively, which delays the treatment of vasculitis. The majority of the strokes caused by TA are ischemic strokes, mostly due to multiple and severe stenosis or occlusive lesions in the aortic arch and its major branches. Couture and colleagues (19) evaluated the impact of stroke on the prognosis of TA patients, and after a median of seven years of follow-up, 59% of the patients had neurological deficits, 35% suffered from stroke recurrence and 24% had epilepsy. However, cerebral aneurysms and subarachnoid hemorrhage are rarely observed in TA patients. The incidence of aneurysm in TA patients is no higher than that in the general population. However, when such incidents occur, aneurysms in TA patients have a high rate of multiplicity and commonly occur in the vertebrobasilar arterial system (20). Posterior reversible encephalopathy syndrome (PRES) is an uncommon neurological complication in TA patients, mainly affecting women under the age of 30, presenting as headache, epilepsy, and neurological deficits in most patients. The pathophysiological mechanism has been suggested to be both endothelial injury and hypertension caused by TA, and the use of immunosuppressants such as cyclosporine and tacrolimus may also be a factor in the development of PRES since they can also induce endothelial dysfunction (21).

In addition, rare complications such as Horner's syndrome and intracranial granulomatosis have been reported as initial manifestations of TA. Other unusual neurological manifestations of TA that have been reported include brachial plexus palsy due to axillary artery aneurysm, unilateral sensorineural hearing loss, subclavian stealing syndrome, Moya-Moya syndrome, multiple cranial nerve palsies, cavernous sinus syndrome, and hypertrophic pachymeningitis.

### Giant Cell Arteritis

Giant cell arteritis (GCA) is the most common large vasculitis in Western countries and mainly affects extracranial branches of the carotid artery and/or the aorta and its large arterial branches, usually affecting people over the age of 50 (22).

Neuro-ophthalmological manifestation is the most frequent and serious event among neurological complications of GCA, accounting for 20–28.8% of affected patients (23, 24), with 15% of them suffering from permanent visual loss (25). Visual symptoms may be caused by ischemic optic neuropathy and retinal ischemia, among which anterior ischemic optic neuropathy accounts for the largest proportion.

Cerebrovascular disease is another severe neurological manifestation, occurring in ~5% of all GCA-related neurological complications. According to recent studies, the risk ratio of cerebrovascular accident in patients with GCA vs. non-GCA comparators was 1.40 (26), and the risk of cerebrovascular complications was higher during the first year (or month) after GCA diagnosis (27, 28), which means that cerebrovascular events often occur during the active period of the disease. Epidemiological study shows that stroke is observed in 1–3% of patients (25), among which cerebral infarction is the most common (58%), followed by subarachnoid hemorrhage (24%), and cerebral hemorrhage (18%) (29). The main reasons are considered to be, on the one hand, ischemia or occlusion caused by direct involvement of carotid and vertebrobasilar arteries and, on the other hand, atherosclerotic changes of the vessels caused by chronic inflammation. The incidence of vertebrobasilar artery vascular accident in patients with GCA (35%) has been shown to be higher than that of the general population (which is usually <15%) (27, 28), and one study showed that audiovestibular dysfunction is not uncommon in GCA patients (30). It is interesting to note that in a retrospective study (25), researchers found that most GCA patients who experienced a vertebrobasilar stroke had neurological symptom onset after the start of corticosteroid treatment. However, it is still being debated whether this situation is due to a direct effect of vasculitis or vascular occlusion promoted by the effects of corticosteroids.

Peripheral nervous system involvement was found in 1–14% of GCA patients, presenting as cranial neuropathies, multiple mononeuropathy, or polyneuropathies (24); existing case reports include multiple cranial nerve palsy, trigeminal autonomic cephalalgia, occipital neuralgia, Horner syndrome, peroneal nerve palsy, cervical radiculopathy, brachial plexopathy, hypertrophic pachymeningitis, and cavernous sinus syndrome.

## Medium-Vessel Vasculitis

Medium vessels refer to the main visceral arteries and veins and their initial branches. Medium-vessel vasculitis consists of polyarteritis nodosa and Kawasaki disease.

### Polyarteritis Nodosa

Polyarteritis nodosa (PAN) is a rare systemic necrotizing vasculitis mainly involving medium and small vessels, causing microaneurysm, stenosis, and thrombosis, therefore leading to ischemia or hemorrhage of the supplied tissues. PAN affects women more often than men, usually with an onset between 40 and 50 years of age (31). The condition may affect any organ; however, the most frequently affected organs are the peripheral nerves, followed by muscles, joints, kidneys, skin, gastrointestinal tract, and heart (32).

Involvement of the CNS has been reported to exist in 20–40% of PAN cases (33) and mainly occurs at late stages (2–3 years) (34). CNS involvement has been recognized as a sign of a poor prognosis, and the expected mortality rate at 5 years is 26% (35, 36). The most common manifestation of CNS involvement includes diffuse encephalopathy and a deficit of focal neurological function. Diffuse encephalopathy may present as new onset seizure and headache, reduced level of consciousness or altered vision, among which some of the cases also suffer from reversible encephalopathy syndrome (37–39). The main manifestations of focal CNS involvement include cerebral infarction (which occurs in 13–17% of PAN patients), hemorrhage, multifocal encephalopathy and episodes of neurological dysfunction that mimic multiple sclerosis (33, 40). Intracranial hemorrhage is a rare complication of PAN, and subarachnoid hemorrhage and intracerebral hemorrhage caused by aneurysms have been reported in PAN cases. The features of these aneurysms were found to be multiple, small in size, and equally located in the infratentorial and supratentorial arteries (41).

Reichart et al. (33) investigated early lacunar strokes complicating PAN and revealed that 33.3% (5/15) of strokes occurred within 1 month after the onset of PAN and 15% (2/13) shortly after the initiation of corticosteroids; ischemic strokes occurred during corticosteroid treatment in 77% (10/13) of the patients, 80% (8/10) of which occurred within 6 months after corticosteroid initiation and 50% (5/10) within 3 weeks. Combined with the results of prior studies showing that all strokes occurred while the patients were under adequate corticosteroid treatment, it was suggested that the promoting effect of corticosteroids in stroke might work by two mechanisms (33). For one thing, corticosteroids promote platelet aggregation in cerebral medium-sized arteries by increasing thromboxane A2, quickly causing a lacunar stroke. Lacunar strokes appearing 1 or more months after initiation of corticosteroids can be explained by the promoting effect of corticosteroids on thrombotic microangiopathies.

PNS involvement is the most common complication of PAN and occurs in 60–70% of cases; it has an onset early in the course of the disease, mostly within a few months of diagnosis (42). The common patterns of PNS involvement include mononeuropathy, polyneuropathy and mononeuritis multiplex, the pathophysiology of which has been recognized as vasculitis of the vasa nervorum. Sudden onset asymmetrical sensorimotor mononeuritis multiplex, which mainly affects the lower extremities, and insidious symmetrical peripheral neuropathy are typical and common in PAN (31). In addition, patients with PAN may also develop pachymeningitis (43) and present with headache or cranial nerve palsy. Sudden bilateral hearing loss (44) and visual alterations caused by optic neuropathy (45) have also been reported.

### Kawasaki Disease

Kawasaki disease (KD) is a systemic vasculitis that mainly affects medium-sized vessels, occurring predominately in children under 5 years of age. Neurological complications of KD have been described in children, such as encephalopathy, seizures, cerebral infarction, intracranial hemorrhage, ataxia, and cranial nerve palsy (46). However, although rare cases of first onset have also been reported in adult patients, most reported cases are in young adults and the manifestations are due to late-onset sequelae instead of new-onset active disease. We found one case (47) describing a 20-year-old young adult with a history of KD suffering from subarachnoid hemorrhage due to an intracranial aneurysm with a stalk-like narrow neck, located at the trunk of the middle cerebral artery. As the researchers mentioned, coronary aneurysms in KD also arise in places where no branch exists. Therefore, non-bifurcation intracranial aneurysm might be related to KD and might cause intracranial hemorrhage in young adults as a late sequela.

### Small-Vessel Vasculitis

Small-vessel vasculitis refers to necrotizing inflammation in the wall of small intraparenchymal arteries, arterioles, capillaries, and venules, and to a secondary degree in medium-size arteries. Vascular and perivascular inflammation leads to fibrinoid necrosis and consequently vascular necrosis, occlusion and thrombosis (10). The disease consists of two major groups, ANCA-associated vasculitis and immune complex small-vessel vasculitis.

### ANCA-Associated Vasculitis

ANCA-associated vasculitis is defined as necrotizing small-vessel vasculitis with few or no immune deposits, mostly associated with myeloperoxidase (MPO) ANCA or proteinase 3 (PR3) ANCA (10). Among the cases of ANCA-associated vasculitis, CNS involvement is more common in granulomatosis with polyangiitis (GPA) and microscopic polyangiitis (MPA) than it is in eosinophilic granulomatosis with polyangiitis (EGPA) (48). Nevertheless, peripheral nerve involvement is more prevalent in EGPA than are MPA and GPA (49).

#### Granulomatosis With Polyangiitis (Wegener's)

Granulomatosis with polyangiitis (GPA) is characterized by necrotizing granulomatous inflammation usually associated with antibodies against PR3 and most commonly involves the upper and lower respiratory tract, and sinonasal inflammation and necrotizing glomerulonephritis are frequently present (10). Neurological complications have been shown to be present in 29–50% of all cases (50–52).

CNS involvement occurs in 7–11% of GPA patients (50, 53, 54), and when isolated cranial nerve palsies are included, the rate is 8–28% (51, 54). Since GPA can commonly involve sinonasal structures and cause granulomatous inflammation, which is capable of invading neighboring structures, the orbit, mastoid, optic nerve, chiasma, cranial nerves, meninges, and pituitary gland might be compromised, presenting as oculomotor dysfunction, mastoiditis, visual disturbances, cranial nerve palsy, headache, and endocrine dysfunction. In addition, small and medium arteries in the cranium can be involved, causing ischemia, or hemorrhage in different vascular territories (55). Cranial nerve palsy was reported to occur in 4.7–6% of GPA patients, among which the most commonly affected cranial nerves are II, VI, and VII (50, 51). The pituitary has been reported to be affected in 1.1–1.3% of GPA patients (56, 57). Meningeal pachymeningitis usually occurs i4n the early stages of GPA, with lower incidences of accompanying systemic symptoms (58), which is perhaps due to granulomatous erosion of the skull base in the early phase and may lead to a delay of diagnosis (52). The most common symptom is headache, followed by cranial nerve palsy and symptoms due to venous sinus occlusion. Vasculitis involvement can cause ischemic and hemorrhagic complications of the brain and spinal cord (58). Dysfunction of intracranial arteries may cause PRES, presenting as various symptoms (59–62). Hypophysitis has been widely reported as one of the CNS complications of GPA, and its clinical manifestations vary according to the specific sites involved. Panhypopituitarism might occur as a consequence of granulomatous inflammation of both the anterior and posterior pituitary (56). Anterior pituitary involvement may influence the release of antidiuretic hormone and present as diabetes insipidus, which was shown to occur in 47 of 58 GPA patients (81%) with sellar involvement, according to an analysis of cases performed by Peters et al. (63). Posterior pituitary inflammation may cause diminished secretion of many hormones, leading to secondary hypogonadism (occurred in 32 out of 58 sellar involved GPA patients, 55%), secondary hypothyroidism (20 out of 58 sellar involved GPA patients, 34.5%), secondary adrenal insufficiency, secondary growth hormone deficiency (19 of 58 sellar involved GPA patients, 32.8%). Compression of the pituitary stalk can result in hyperprolactinemia (5 of 58 sellar involved GPA patients, 8.6%) and galactorrhea, and visual alteration may occur if the optic chiasm is compressed (56, 57).

PNS complications have been reported to occur in 11–44% of GPA patients (54), which occurs milder and later in the course of the disease than in other ANCA-associated vasculitis (64), presenting mainly as recurrent mononeuropathies, mononeuritis multiplex, or symmetric polyneuropathy due to ischemia caused by vasculitic inflammation of the vasa nervorum. As the most frequent manifestation, distal symmetrical sensory neuropathy was reported in 7–10% of cases, followed by motor mononeuritis multiplex in 2–12% (51, 53). Commonly, peroneal (90–95%), tibial (38–55%), ulnar (35–45%), and median (26–36%) nerves are involved (29, 51).

#### Eosinophilic Granulomatosis With Polyangiitis (Churg-Strauss)

The characteristics of eosinophilic granulomatosis with polyangiitis (EGPA) is an eosinophil-rich and granulomatous inflammation that often involves the respiratory tract and is associated with asthma and eosinophilia (10).

Neurological involvement is common in EGPA, accounting for up to 60% of cases and usually manifesting as peripheral neuropathy that tends to present before visceral involvement (29). Therefore, early detection of peripheral nerve involvement is fundamental for early diagnosis and treatment of EGPA. The most common peripheral neurological manifestation is multiple mononeuropathy (accounting for 68% of cases with peripheral neuropathy), followed by distal symmetric polyneuropathy (28%) and asymmetric polyneuropathy (4%) (65). Since mononeuritis multiplex is typical in acute systemic vasculitis, the initial symptoms commonly present as dysesthesia, paresthesia, and edema in distal limbs, especially the lower limbs. The condition will gradually progress into asymmetrical polyneuropathy and may affect motor nerves, leading to muscle atrophy. Electrophysiological studies have shown decreases in the amplitude of sensory nerve action potentials and compound muscle action potentials, indicating the presence of axonal injury (66).

CNS involvement in EGPA is rather rare, accounting for only 6–10% of all cases (67). Cerebral infarctions and intracerebral hemorrhage resulting from intracranial vasculitis are the most common CNS presentations of EGPA. In addition, rarer manifestations have been noted, such as subarachnoid hemorrhage, cranial nerve palsy, encephalopathy, epilepsy, hydrocephalus, headache, sinus venous thrombosis, spinal hemorrhage, meningeal involvement, and optic neuropathy.

#### Microscopic Polyangiitis

Microscopic polyangiitis (MPA) presents with non-granulomatous inflammation with few or no immune deposits in the walls of the affected vessels, mainly associated with antibodies against MPO. Necrotizing glomerulonephritis and alveolar hemorrhage are common complications (10). Peripheral neurological involvement occurs in 55 to 79% of cases with MPA, with no special manifestations compared with those of other ANCA-associated types of vasculitis (mainly presenting as polyneuropathy and mononeuropathy); (64). CNS involvement in MPA is rare and was reported to be able to manifest as intracerebral infarction or hemorrhage, subarachnoid hemorrhage, hypertrophic pachymeningitis, PRES or spinal cord involvement (68).

### Immune Complex Small-Vessel Vasculitis

Immune complex small-vessel vasculitis is characterized by moderate to marked deposits of immunoglobulin and/or complement components in the small vessel walls.

#### Antiglomerular Basement Membrane Disease

Antiglomerular basement membrane (anti-GBM) disease, also called Goodpasture syndrome, is a vasculitis affecting glomerular capillaries and/or pulmonary capillaries with deposition of anti-GBM autoantibodies on GBM, accounting for 5% of adult patients with glomerulonephritis and leading to acute renal failure in approximately half of the patients. Lung involvement results in pulmonary hemorrhage, and renal involvement leads to glomerulonephritis with necrosis and crescents, which are the classic presentations of anti-GBM disease (10, 69). Neurological involvement is uncommon. According to the rare cases reported (69–73), PRES is the most frequent neurological complication. All patients reported are younger than 40 and they all manifested as hypertension, renal failure, and seizures. Destruction of the blood-brain barrier resulting from inflammatory endothelial injury and increased capillary filtration due to hypertension are important factors in the pathophysiology of PRES in GBM patients.

#### Cryoglobulinemic Vasculitis

Cryoglobulinemic vasculitis (CV) is defined as vasculitis with cryoglobulin immune deposits affecting small vessels (predominantly capillaries, venules, or arterioles) caused by chronic inflammation, autoimmune disorders, and lymphoproliferative disorders, frequently involving skin, glomeruli, and peripheral nerves (10). Most cases of cryoglobulinemic vasculitis result from infection, B-cell lymphoproliferative disorders and autoimmune diseases, among which hepatitis C virus infection is the cause in ~80% of cases (74). In a study of 242 cases with non-infectious mixed cryoglobulinemia vasculitis (75), 117 patients (48%) were found to have no identified causal factor, also defined as idiopathic. However, no study has evaluated the neurological involvement of idiopathic cryoglobulinemia vasculitis alone. For patients with mixed CV, PNS involvement is not uncommon and typically starts with polyneuropathy that affects the sensory nerves initially and later the motor nerves, predominantly involving the lower limbs. CNS involvement is rare, and studies have shown that mixed CV may cause cerebrovascular events, PRES, hydrocephalus and intracranial hypertension (29, 76).

#### IgA Vasculitis (Henoch-Schönlein Purpura)

IgA vasculitis (IgAV) is characterized by IgA1-dominant immune deposits affecting small vessels, often involving the skin and gastrointestinal tract and frequently causing arthritis (10). The majority (75%) of cases occur in young people no more than 20 years of age (77), and CNS involvement has been reported to manifest as headache, decreased consciousness, seizures, focal neurological deficits, and visual and verbal abnormalities. However, PNS involvement has been documented as neuropathies of the brachial plexus and facial, peroneal, femoral and ulnar nerve, and in addition, Guillain–Barre syndrome and mononeuritis multiplex (78). Rare cases of IgAV-induced neurological manifestations have been reported in adult patients as ischemic stroke, axonal sensorimotor polyneuropathy (79, 80), mononeuritis multiplex (81, 82), acoustic neuritis, facial nerve palsy (83), intracerebral hemorrhage (84), anterior ischemic optic neuropathy (85), focal seizures (86), and encephalopathy (87).

#### Hypocomplementemic Urticarial Vasculitis (anti-C1q Vasculitis)

Hypocomplementemic urticarial vasculitis (HUV) is a kind of vasculitis accompanied by urticaria and hypocomplementemia affecting cutaneous small vessels and is associated with anti-C1q antibodies, commonly causing urticaria and multiorgan involvement such as glomerulonephritis, arthritis, obstructive pulmonary disease, and ocular inflammation (10). HUV may be induced by connective tissue diseases, infections (such as hepatitis B, hepatitis C and infectious mononucleosis), neoplasms and drugs; however, most cases of HUV are idiopathic (88). Neurological involvement has been occasionally reported as pseudotumor cerebri (89), lower cranial nerve (VIII, IX, and X) palsies (90) and peripheral nerve involvement such as asymmetrical multifocal axonal sensory neuropathy.

## Variable-Vessel Vasculitis

Variable-vessel vasculitis refers to vasculitis with no predominant type of vessel involved that can affect vessels of any size (small, medium, and large) and type (arteries, veins, and capillaries) (10).

### Behçet's Syndrome

Behçet's syndrome is a vasculitis occurring in patients with Behçet's disease (BD) and can affect arteries or veins and lead to a relapsing inflammatory disorder in almost any tissue, with oral and genital ulcerations and uveitis as its most typical symptoms (41). Neurological manifestations of BD, which are called neuro-BD (NBD), occur in 1.3–59% of all cases and usually affect patients aged between 20 and 40 years, with a male predominance (the male to female ratio is 2.8:1) (91–95). NBD commonly appears 3–6 years after other systemic involvement (92, 96, 97) and is considered to be caused by perivasculitis.

The manifestations of neurological involvement can be caused by either CNS parenchymal inflammation or vascular complications in the nervous system, with a reported prevalence of 67–76 vs. 12–20% in all NBD cases (93, 98). The former is considered to be caused by small vasculitis, leading to axonal damage and gliosis, frequently affecting the brain stem (occurs in 25–50% of all parenchymal NBD cases) (92, 93, 99, 100), thalamus and basal ganglia, and, only rarely, the white matter and spinal cord. The main clinical manifestations are subacute cranial neuropathy, ophthalmoparesis, meningoencephalitis, and alteration of cerebellar, pyramidal and extrapyramidal function. Uncommon symptoms such as subcortical dementia, stroke and transverse myelitis have also been reported (91, 101). Vascular complications mainly result from cerebral venous thrombosis (CVT) due to large vessels endothelial cell activation, and rarely, aneurysms due to perivasculitis to the vasa nervorum (91). CVT in BD mainly causes focal neurological deficits and seizures in young men (102). However, not all epileptic seizures in NBD patients result from CVT, and brainstem lesions may also lead to complex partial seizures (103).

In addition, perivasculitis to the vasa nervorum may also cause PNS involvement. However, this condition is extremely rare and was found in merely 8 out of 1,031 cases (0.8%); thus, its relationship with BD is still doubtful (91).

### Cogan Syndrome

Cogan syndrome (CS) is a sequence of clinical manifestations due to a chronic immunological inflammatory multisystem disease of unknown origin, characterized by recurrent episodes of keratitis, vestibuloauditory dysfunction, and systemic vasculitis, frequently leading to visual loss, vertigo, sensorineural hearing loss, tinnitus and systemic manifestations (10). The syndrome mainly affects children and young adults with an average age at onset of 38 years (SD, 15.1 years; range, 9–70 years) (104).

It is worth noting that complications of the eyes and ears do not always occur at the same time (sometimes up to 9 years apart) (105) and in 20% of cases, vestibuloauditory symptoms can be the only presentation (104). Therefore, although the vestibuloauditory involvement in Cogan syndrome is mainly due to inflammation of inner ear structures rather than neurological involvement (105), its manifestations can be easily misdiagnosed as central acute vestibular syndrome of vascular origin, Ménière's disease or vestibular migraine, which are common in neurological out-patients. According to a study that enrolled 60 cases of CS at Mayo Clinic (104), all cases suffered from hearing loss, 90% of the cases experienced vertigo, and tinnitus (80%), ataxia (53%), and oscillopsia (25%) were also common during the course of the disease. Hearing loss is typically sudden, mainly involves bilateral high frequencies, and the decline in hearing fluctuates and progresses gradually. Vestibular function is commonly damaged bilaterally (accounting for 74% of cases that underwent a caloric test) and vestibular symptoms may last for days to weeks (sometimes indefinitely) with no resolution (104, 106). The vestibuloauditory symptoms listed above may recur in 1–13 years after onset (occurs in 22% of patients) (107).

Neurological involvement was reported in 29–56% of cases and usually occurs after eye and ear manifestations (106, 108). CNS involvement may manifest as ischemic stroke (accounting for 2.5–3% of cases reported), encephalitis (5–6%), meningitis (5–22%), encephalopathy, myelopathy, optic nerve disorders, aneurysm, and cerebral venous thrombosis. PNS conditions in Cogan syndrome patients have been reported as peripheral neuropathy (1–12.5%, frequently mononeuritis multiplex), cranial neuropathy (1–10%, mainly cranial nerve II, V, VI, and VII) and myopathy (106).

## Conclusion

Neurological involvement is a common complication of PSV (Table 1), and neurologists play an important role in the identification and diagnosis of PSV patients with otherwise unexplained neurological symptoms as their chief complaint. This article summarizes the neurological manifestations of PSV and hopes to improve neuroscientists' understanding of this broad range of diseases.

**Table 1:** Common CNS and PNS involvements of primary systemic vasculitis.

**PSV**	**CNS involvement**	**PNS involvement**
Takayasu arteritis	Dizziness (78.1%), headache (25.5%); visual disturbances (4.6–59.3%); syncope (4–21.9%); stroke (10–20%);	Rare
Giant cell arteritis	Neuro-ophthalmological damage (20–28.8%), stroke (1–3%), vertebrobasilar artery vascular accident (35%);	1–14% of cases; cranial neuropathies, multiple mononeuropathy, polyneuropathies;
Polyarteritis nodosa	20–40% of cases; Diffuse encephalopathy, cerebral infarction (13–17%);	60–70% of cases; Mononeuropathy, polyneuropathy, mononeuritis multiplex;
Granulomatosis with polyangiitis	8–28% of cases; Cranial nerve palsy (4.7–6%, mainly II, VI, and VII), pituitary damage (1.1–1.3%), meningeal pachymeningitis, ischemic, and hemorrhagic complications of brain and spinal cord, PRES	11–44% of cases; Recurrent mononeuropathies, mononeuritis multiplex, symmetric polyneuropathy
Eosinophilic granulomatosis with polyangiitis	6–10% of all cases; Cerebral infarctions and intracerebral hemorrhage	~60% of cases; Multiple mononeuropathy (68% of PNS cases), distal symmetric polyneuropathy (28%) and asymmetric polyneuropathy (4%)
Microscopic polyangiitis	Rare	55–79% of cases; polyneuropathy, mononeuropathy
Behçet's syndrome	CNS parenchymal inflammation (67–76% of all NBD cases): subacute cranial neuropathy, ophthalmoparesis, meningoencephalitis, alteration of cerebellar, pyramidal, and extrapyramidal function; Vascular complications in the nervous system (12–20% of all NBD cases): cerebral venous thrombosis, aneurysms	Extremely rare: 0.8% of BD cases
Cogan syndrome	Ischemic stroke (2.5–3%), encephalitis (5–6%), meningitis (5–22%), encephalopathy, myelopathy, optic nerve disorders, aneurysm, and cerebral venous thrombosis	Peripheral neuropathy (1–12.5%), cranial neuropathy (1–10%, mainly II, V, VI, and VII) and myopathy

## Author Contributions

SZ conceived the article and wrote the manuscript. DY and GT reviewed and edited the manuscript. All authors read and approved the manuscript.

### Conflict of Interest Statement

The authors declare that the research was conducted in the absence of any commercial or financial relationships that could be construed as a potential conflict of interest.
